# Working from home: mismatch between access and need in relation to work–home interference and fatigue

**DOI:** 10.5271/sjweh.3983

**Published:** 2021-10-31

**Authors:** Astrid de Wind, Debby GJ Beckers, Hylco H Nijp, Wendela Hooftman, Angela GEM de Boer, Sabine AE Geurts

**Affiliations:** 1 Amsterdam UMC, University of Amsterdam, Department of Public and Occupational Health, Coronel Institute of Occupational Health, Amsterdam Public Health research institute, The Netherlands; 2 Radboud University, Behavioural Science Institute, Nijmegen, The Netherlands; 3 HAN University of Applied Sciences, Academy of Organization and Development, Nijmegen, The Netherlands; 4 Netherlands Organization for Applied Scientific Research TNO, Leiden, The Netherlands

**Keywords:** remote work, well-being, work location flexibility, worktime control

## Abstract

**Objectives:**

Working from home (WfH) is a promising practice that may enable employees to successfully and sustainably combine work and private life. Yet, not every employer facilitates WfH and not every employee has similar needs concerning the practice. The current study aims to examine the association of a WfH mismatch with work–home interference (WHI) and fatigue.

**Methods:**

Data on WfH, WHI, and fatigue of a quasi-representative sample of 2374 Dutch employees in 2012/13 and a follow-up measurement one year later were used. Cross-sectional and longitudinal regression analyses were conducted to investigate the cross-sectional and temporal associations between WfH mismatch on the one hand and (changes in) time-based and strain-based WHI and fatigue on the other hand.

**Results:**

In the cross-sectional analyses, WfH mismatch was significantly associated with higher time-based WHI (B=0.13), strain-based WHI (B=0.17) and more fatigue (B=0.32). WfH mismatch was not associated with changes in these outcomes after one year of follow-up.

**Conclusions:**

A tailored WfH organizational policy, in which employees’ need for working from home is taken into account, may be a fruitful approach to utilize WfH as a way for employees to successfully and sustainably combine work and private life to its full potential.

Three parallel societal and labor market developments have greatly challenged European workers in recent years. First, in most European countries, the workforce is ageing (1). To keep the pension system affordable, many governments have implemented measures to encourage prolonged working, eg, by raising the statutory retirement age. A second development is the rise in the number of double-income families: in Europe female employment increased from 58.1 to 68.2% in the period 2002–2019 (2). Although increased labor participation among women has important benefits, a taxing side-effect is that workers have to combine paid work with household and care responsibilities more often. A third development is the increasing demand governments place on people, including working people, to (also) provide informal care for relatives (3).

These three developments, both in the workforce and society as a whole, challenge an appropriate balance between effort and recovery and between working life and private life. A lasting situation of expending high effort and having too little time and opportunities for recovery is a serious risk factor for ill-health and reduced quality of life (4). Combining high work demands with high responsibilities in private life poses a risk for negative work–home interference (WHI), ie, “when work demands absorb time and/or create strain that makes adequate functioning in the family domain more difficult” (5). In the long term, these two related types of imbalance can result in stress-related health problems, such as burnout, depression and substance abuse (6). As such, the three societal and labor market developments make healthy working conditions, which enable people to work sustainably, increasingly important.

The possibility to work (partly) from home, in the literature – also referred to as work location flexibility, remote work or spatial flexibility – may be a promising way to enable employees to successfully and sustainably combine working and private life, while keeping an appropriate balance between effort and recovery. Working from home (WfH) may, for example, enable employees to bring their children to school in the morning. Flexibility in work location may also be attractive for employees to reduce commuting time, leaving more time for actual work or meeting private life responsibilities. These factors may (partly) explain why working remotely has gained in popularity. In fact, in Europe the proportion of workers who sometimes or usually work from home increased from 12.4% in 2008 to 16.1% in 2019 (7). In The Netherlands, this proportion is even higher, with an increase in prevalence from 27.3% in 2010 (8) to 36.9% in 2019 (9). The current COVID-19 pandemic, especially the social distancing measures that governments have been forced to take, have further catalyzed this development and will continue to do so after the COVID-19 pandemic has passed (10).

The prevalence of WfH is not equally distributed across sectors, with logically sectors that require a fixed workplace (with on-site work responsibilities, such as in the healthcare and hospitality sector) showing a lower prevalence than those with work tasks that can be performed remotely (11). In The Netherlands, working from home is most common in financial services (72.6%), information and communication technology (72.0%) and education (67.9%) (9). During the COVID-19 pandemic, many organizations had to facilitate working from home out of necessity. Although the long-term impact of the COVID-19 pandemic on the organization of work remains to be seen, working from home rates are likely to increase (10).

WfH can be theorized to have favorable effects on WHI and health through two mechanisms that relate to worktime control, ie, “an employee’s perception of his/her possibilities to control the duration, position, and distribution of his/her working times” (12). First, employees who work from home or on another preferred location may be better able to regulate time demands. As such, work location flexibility can function as a *time-regulation mechanism*. It enables employees to adapt working times to responsibilities in private life. This time-regulating function of WfH may protect employees from time-based WHI (ie, having difficulty fulfilling demands at home due to time devoted to work) (13). Two meta-analyses indeed showed a statistically significant (albeit weak) relationship between WfH and lower work-home conflict (14, 15). A second mechanism is that WfH enables the employee to adjust working and resting times to momentaneous recovery needs, ie, a *recovery-regulation mechanism*. The recovery-regulating function of WfH may protect employees for work-related fatigue and strain-based WHI (ie, having difficulty fulfilling demands at home due to strain built up at work) (13).

Despite these two general mechanisms regarding the overall favorable effects of WfH on WHI and fatigue, it is relevant to consider that not every employee will have a similar need for or access to WfH. Also, need for and access to WfH may not always match, resulting in a WfH mismatch. Individual differences in need for WfH may be caused by individual differences in integration and segmentation preferences [eg, (16)]. Employees with a segmentation preference want to separate the work and home domains as much as possible, whereas employees with an integration preference desire to integrate these domains (16). As such, the second group is likely to experience a higher need for WfH than the first. Also, not every employer permits and facilitates their employees to work from home, resulting in differences between employees’ access to WfH. Based on the theoretical notion of person–environment fit that individual well-being is optimized when individual preferences and needs fit contextual factors, it can be assumed that well-being outcomes among employees are optimal when their need for WfH (individual characteristic) on the one hand and their access to WfH (contextual factor) on the other hand are well connected (17).

Previous research on the health effects of WfH did not appropriately distinguish between workers’ need for and access to WfH and thus, cannot appropriately study the implications of a WfH mismatch for workers’ health and wellbeing. A previous study of Nijp et al (18) was the first to follow this reasoning for the related concept of worktime control. The study showed that over 40% of day workers experienced a mismatch between need for and access to worktime control, and that such a mismatch was associated with a higher level of WHI and fatigue. To date, no studies have focused on the association between WfH need versus access mismatch on the one hand and WHI and fatigue on the other.

To address this knowledge gap, the current study aims to examine the relationship of an unfavorable mismatch between need for and access to WfH [ie, need for WfH > access to WfH (ie, WfH mismatch)] with time-based and strain-based WHI and fatigue. Based on the time- and recovery-regulation mechanisms described above and in line with the theoretical notions of individual differences and person–environment fit, we hypothesize a WfH mismatch to be associated with higher levels of time- and strain-based WHI and work-related fatigue.

## Methods

To study the association of WfH mismatch with WHI and fatigue, the current study builds on Nijp et al’s previous study (18), which investigated the association of a work time control mismatch with WHI and fatigue. To allow comparison, the methods of the present study were matched with those of this previous study as much as possible with the exception that the previous study included only cross-sectional data, whereas the current study also includes data from a one-year follow-up questionnaire.

### Dataset and study sample

For the current study, we used data that were collected among a sample of the participants of The Netherlands Working Conditions Survey (NWCS) in 2010 (N=23 788) (19). NWCS is a periodical survey among employees with a broad range of occupational backgrounds and aims to monitor quality of work and employment in The Netherlands. All followed procedures concerning NWCS are in accordance with the Helsinki Declaration as revised in 2008. Potential respondents of NWCS were informed about the study in a letter accompanying the questionnaire. Participation in the questionnaire was considered informed consent. All data was pseudonymized before access was provided to the researchers.

Respondents who participated in NWCS 2010 and who had given permission to be approached again, were invited for participation in a new questionnaire in autumn 2012 (N=5504). The response rate was 48% (2012; N=2633). After one year, 53% of this population also participated in a second follow-up questionnaire (2012 and 2013; N=1393). Another 7% only participated in this follow-up questionnaire (only 2013; N=405).

**Figure 1:**
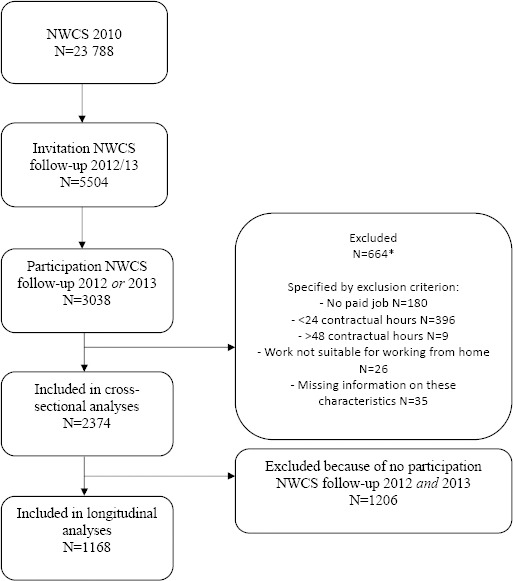
Flow of the study sample. * Numbers related to specific exclusion criteria do not add up to the total number of exclusions because people may be excluded for several overallping critera.

To examine the relationship of WfH mismatch with time- and strain-based WHI and fatigue, we used a cross-sectional sample including participants who participated in either the 2012 or 2013 measurement or both. Having a paid job was an inclusion criterion. Participants who reported that their work did not lend itself for WfH were excluded. Furthermore, workers who had <24 or >48 contractual hours/week were excluded. We considered working 24 hours/week as a minimum: sufficient exposure in terms of work hours/week is important in a study on WHI and work-related fatigue. Working 48 hours/week was considered the upper limit: this is the legal maximum work hours/week (on average in a 16-week period) in The Netherlands (20). These inclusion and exclusion criteria resulted in a study sample of 2374 persons for the cross-sectional analyses. To replicate cross-sectional findings also longitudinally, we additionally applied analyses on the sample of respondents who participated in the 2012 and the 2013 measurement (N=1168) (figure 1).

### Measures

Information on WfH, time- and strain-based WHI, fatigue and control variables were derived from the NWCS follow-up questionnaires in 2012 and 2013.

*WfH mismatch* has been operationalized based on self-developed items on WfH need and access. *WfH need* was assessed with the following question: “To what extent do you have the need to work from home one or more days per week?” *WfH access* was assessed with the following question: “To what extent do you have the possibility to work from home?”. A 5-point Likert scale was used ranging from “almost not” (1) to “to a very strong degree” (5). WfH need and access were dichotomized. The answers “almost not” and “to a limited degree” were classified as low, and the answers “to a reasonable degree” until “to a very strong degree” were classified as high. A mismatch variable was created by subtracting respondents’ need scores from their access scores. Respondents were categorized into a match group (including those with a positive mismatch), ie, access >≈ need (values -1, 0, 1, 2, 3 or 4) or an unfavorable mismatch group, ie, access < need (values -4, -3 or -2).

*WHI* has been operationalized with six items derived from two sub-scales from the Nijmegen Work-Home Interaction Survey (21), ie, *time-based WHI* and *strain-based WHI*. An example item of time-based WHI is: “How often does it happen that your work schedule makes it difficult for you to fulfil your domestic obligations?” An example item of strain-based WHI is: “How often does it happen that your work obligations make it difficult for you to feel relaxed at home?” A 4-point Likert scale was used raging from “almost never” to “almost always”. Mean scores were calculated for both sub-scales based on the individual items. Higher scores indicate higher levels of time-based WHI and strain-based WHI. Cronbach’s alpha’s were 0.77 and 0.79 for time-based and strain-based WHI, respectively.

*Fatigue* was measured using four items from the Fatigue Assessment Scale (eg, “I have enough energy for everyday life”) (22) and one item from the Questionnaire for Experience and Assessment of Work (ie, “In general, I only start to feel relaxed on the second non-working day”) (23). A 5-point Likert scale was used ranging from “almost never” to “almost always”. One item was mirrored and, as such, had to be reverse coded. A mean score was calculated for fatigue based on the five individual items (including the reverse coded item). Higher scores indicate higher levels of fatigue. Cronbach’s alpha was 0.82 for fatigue.

*Age*, *sex* and *educational level* were included as control variables. Educational level was measured using a question on the highest level of education completed with a diploma, and categorized into low (primary school, lower and intermediate secondary education or lower vocational training), intermediate (higher secondary education or intermediate vocational training), and high (higher vocational education or university).

*Motives to (not) work from home* were included to gain insight in why employees choose to use or not to use the possibility to work from home. Respondents who used the possibility to work from home were asked to indicate from a list of eight possible motives, which ones applied to them. Respondents who did not use the possibility to work from home could choose from a list of seven possible motives. These variables are not included in the main analyses but are used to provide insight in the most common reasons to use or not to use the possibility to work from home within the study population.

### Analyses

Descriptive statistics, ie, means, standard deviations (SD), frequencies and percentages, were used to report on baseline characteristics and the three most-frequently selected reasons to use or not to use the possibility to work from home. Frequency analyses were conducted to examine the prevalence of WfH mismatch.

Regression analyses were conducted to investigate the association between WfH mismatch on the one hand, and time-based WHI, strain-based WHI and fatigue, on the other hand. All models were adjusted for age, sex and educational level. Firstly, the analyses were conducted with cross-sectional data. To test whether there were differences between male and female, we included an interaction term between WfH mismatch and sex in these analyses. Thereafter, the analyses were conducted with a longitudinal design in which we examined the temporal association between WfH mismatch in 2012 and WHI and fatigue in 2013, controlling for the levels of WHI and fatigue in 2012. This implies that we investigated the relations between WfH mismatch in 2012 with changes in levels of WHI and fatigue in the following year.

## Results

Table 1 shows descriptive statistics of control variables and main study variables of the sample used for cross-sectional analyses for the total group as well as separately for the WfH match and mismatch groups. The average age of the study sample was 44.2 years. Most participants were male (62.7%), and had an intermediate (37.9%) or a high (52.7%) educational level. Respondents were mainly working in business services (18%), healthcare (15%), industry (14%), education (12%), public administration (12%) and trading (10%).Respondents reported, on average, a score of 2.5 for WfH need, and 46.1% of the participants were classified to have high WfH need (table 1). On average, respondents scored 2.1 for WfH access, and 32.1% could be classified as having high WfH access. With regard to WfH mismatch 21.1% of the participants were classified as having a WfH mismatch (access < need). Table 2 shows descriptive statistics of control variables and main study variables (at baseline and follow-up) of the sample used for the longitudinal analyses. WfH mismatch was stable over time, with 85% remaining either in the match group or in the mismatch group between baseline and follow-up, 9% changing from match to mismatch and 6% changing from mismatch to match (data not shown in table). Also, the outcomes time-based WHI, strain-based WHI and fatigue were stable over time. When applying a dichotomous operationalization, 14% changed from low to high time-based WHI and 10% from high to low, 16% changed from low to high strain-based WHI and 9% from high to low, and 12% changed from low to high fatigue and 12% from high to low (data not shown in table).

**Table 1:** Descriptive statistics of control variables and main study variables of the sample used for cross-sectional analyses. [SD=standard deviation; WfH=working from home.]

Variable	Total group (N=2374)		Match group (N=1759)		Mismatch group (N=471)	
		
	Mean (SD)	N ^a^ (%)	Mean (SD)	N ^a^ (%)	Mean (SD)	N ^a^ (%)
Age	44.2 (11.2)		44.6 (11.1)		43.6 (11.2)	
Sex						
Male		1489 (62.7)		1117 (63.5)		277 (58.8)
Female		885 (37.3)		642 (36.5)		194 (41.2)
Educational level						
Low		222 (9.4)		153 (8.7)		46 (9.8)
Intermediate		900 (37.9)		659 (37.5)		167 (35.5)
High		1250 (52.7)		946 (53.8)		258 (54.8)
WfH need	2.5 (1.5)		2.1 (1.3)		4.1 (0.8)	
Low		1203 (53.9)		1203 (68.4)		0
High		1027 (46.1)		556 (31.6))		471 (100)
WfH access	2.1 (1.3)		2.3 (1.4)		1.5 (0.7)	
Low		1592 (67.9)		1075 (61.1)		428 (90.9)
High		753 (32.1)		684 (38.9)		43 (9.1)
WfH use	3.1 (1.2)		3.1 (1.2)		3.1 (1.1)	
Low		270 (36.0)		247 (36.1) ^b^		12 (27.9) ^b^
High		481 (64.0)		437 (63.9)		31 (72.1)
WfH mismatch	-0.4 (1.5)		0.2 (0.9)		-2.6 (0.8)	
Match		1759 (78.9)		NA		NA
Unfavorable mismatch		471 (21.1)				
Time-based work-home interference (1–4)	1.6 (0.6)		1.5 (0.5)		1.7 (0.6)	
Strain-based work-home interference (1–4)	1.6 (0.6)		1.6 (0.5)		1.8 (0.6)	
Fatigue (1–5)	2.2 (0.8)		2.1 (0.8)		2.4 (0.9)	

a For some variables, data were missing for a maximum of 144 persons.

b Use was only asked to participants who had access to working from home.

**Table 2:** Descriptive statistics of control variables and main study variables (at baseline and follow-up) of the sample used for longitudinal analyses. [SD=standard deviation; WfH=working from home.]

Variable	Baseline		Follow-up	
	
	Mean (SD)	N ^a^ (%)	Mean (SD)	N ^a^ (%)
Age	44.0 (10.6)			
Sex				
Male		744 (63.7)		
Female		424 (36.3)		
Educational level				
Low		109 (9.3)		
Intermediate		422 (36.2)		
High		636 (54.5)		
WfH need				
Low		642 (56.7)		553 (49.3)
High		490 (43.3)		568 (50.7)
WfH access				
Low		790 (68.0)		746 (64.4)
High		371 (32.0)		413 (35.6)
WfH use				
Low		139 (37.5)		154 (37.3)
High		232 (62.5)		259 (62.7)
WfH mismatch				
Match		910 (80.4)		863 (77.0)
Unfavorable mismatch		222 (19.6)		258 (23.0)
Time-based work-home interference (1–4)	1.5 (0.6)		1.6 (0.6)	
Strain-based work-home interference (1–4)	1.6 (0.5)		1.7 (0.6)	
Fatigue (1–5)	2.1 (0.8)		2.2 (0.8)	

a For some variables, data were missing for a maximum of 51 persons.

### Motives to (not) work from home

Among the respondents who had access to work from home and additionally made use of this possibility, the three most-frequently selected motives were (i) to be more productive (62.9% of those who responded to this question), (ii) to facilitate a good balance between working life and private life (42.7%), and (iii) to reduce commuting time (37.1%). Among the respondents who had WfH access but did not make use of this possibility, the three most-frequently selected reasons were (i) to promote a proper separation between working life and private life (4.7%), (ii) to facilitate collaboration with colleagues (37.9%), and (iii) to have better (social) contact with colleagues (33.6%).

### WfH mismatch in relation to time-based WHI

Table 3a shows adjusted models resulting from the linear regression analysis for WfH mismatch and time-based WHI using cross-sectional and longitudinal data. Using cross-sectional data, WfH mismatch was associated with a higher level of time-based WHI than a positive mismatch or a match (B 0.13). There was no significant interaction between WfH mismatch and sex (P=0.23) (data not shown in table). Explained variance (R^2^) of WfH mismatch was 0.009 beyond the explained variance of 0.007 attributed to the control variables only. Using the longitudinal data, WfH mismatch was not associated with a change in time-based WHI between 2012 and 2013.

**Table 3a:** Linear regression analyses for time-based work-home interference (WHI) with cross-sectional and longitudinal data.[CI=confidence interval; WfH=working from home.]

	Adjusted model using cross-sectional data ^a^	Adjusted model using longitudinal data ^b^
	
	B (95% CI)	B (95% CI)
WfH mismatch	0.13 (0.07–0.19) ^c^	0.03 (-0.03–0.10)
Age	-0.00 (-0.01–0.00) ^c^	- 0.00 (-0.00–0.00)
Sex (female)	-0.05 (-0.09–0.00) ^d^	-0.06 (-0.11– -0.01) ^c^
Educational level	0.05 (0.01–0.08) ^c^	0.03 (-0.01–0.06)
Time-based WHI at baseline		0.61 (0.57–0.66) ^c^

a Adjusted for age, sex, educational level.

b Adjusted for age, sex, educational level and time-based WHI at baseline.

c P<0.05.

d P<0.10.

### WfH mismatch in relation to strain-based WHI

Table 3b shows adjusted models resulting from the linear regression analyses for WfH mismatch and strain-based WHI using cross-sectional and longitudinal data. Using cross-sectional data, WfH mismatch was associated with strain-based WHI with a higher level of strain-based WHI than a positive mismatch or a match (B=0.17). There was no significant interaction between WfH mismatch and sex (P=0.34) (data not shown in table). Explained variance of WfH mismatch was 0.016 beyond the explained variance of 0.020 attributed to the control variables only. Using the longitudinal data, WfH mismatch was not associated with a change in strain-based WHI between 2012 and 2013.

**Table 3b:** Linear regression analyses for strain-based work-home interference (WHI) with cross-sectional and longitudinal data. [CI=confidence interval; WfH=working from home.]

	Adjusted model using cross-sectional data ^a^	Adjusted model using longitudinal data ^b^

	B (95% CI)	B (95% CI)
WfH mismatch	0.17 (0.12–0.23) ^c^	0.05 (-0.02–0.12)
Age	-0.00 (-0.01– -0.00) ^c^	0.00 (-0.00–0.00)
Sex (female)	-0.01 (-0.06–0.04)	-0.01 (-0.06–0.05)
Educational level	0.10 (0.07–0.14) ^c^	0.05(0.02–0.09) ^c^
Strain-based WHI at baseline		0.68(0.63–0.73) ^c^

a Adjusted for age, sex, educational level.

b Adjusted for age, sex, educational level and strain-based WHI at baseline.

c P<0.05.

### WfH mismatch in relation to fatigue

Table 3c shows adjusted models resulting from the linear regression analyses for WFH mismatch and fatigue using cross-sectional and longitudinal data. Using cross-sectional data, WfH mismatch was associated with more fatigue than a positive mismatch or a match (B 0.32). There was no significant interaction between WfH mismatch and sex (P=0.31) (data not shown in table). Explained variance of WfH mismatch was 0.027 beyond the explained variance of 0.008 attributed to the control variables only. Using the longitudinal data, WfH mismatch was not associated with a change in fatigue between 2012 and 2013.

**Table 3c:** Linear regression analyses for fatigue with cross-sectional and longitudinal data. [CI=confidence interval; WfH=working from home.]

	Adjusted model using cross-sectional data ^a^	Adjusted model using longitudinal data ^b^
	
	B (95% CI)	B (95% CI)
WfH mismatch	0.32 (0.24–0.40) ^c^	0.06 (-0.03–0.15)
Age	-0.01 (-0.01–0.00) ^c^	0.00 (-0.00–0.00)
Sex (female)	0.07 (0.00–0.14) ^c^	0.06 (-0.08–0.14) ^c^
Educational level	-0.04 (-0.09–0.02)	0.00 (-0.05–0.05)
Fatigue at baseline		0.73 (0.69–0.78) ^c^

a Adjusted for age, sex, educational level.

b Adjusted for age, sex, educational level and fatigue at baseline.

c P<0.05.

## Discussion

This study showed that an unfavorable WfH mismatch was associated with higher levels of time- and strain-based WHI and more fatigue in the cross-sectional analyses, but it was not associated with changes in these outcomes in the longitudinal analyses. This implies that a WfH mismatch is associated with higher WHI and more fatigue, but that it is not related to further worsening of WHI and increasing fatigue over time.

Previous research mainly examined WfH access in relation to employee wellbeing. Our findings showed that it is important to not merely consider WfH access, but to take on a richer conceptualization of WfH, including attention for employee preferences and the match between access to and need for WfH. These insights align with the study by Nijp et al (18), showing that a proper match between need for- and access to worktime control may help to prevent problems in work-life balance, fatigue and work motivation. It should, however, be noted that the associations between WfH mismatch on the one hand and time- and strain-based WHI and fatigue on the other hand were rather weak in the current study. Interestingly, our results on motives to work from home or not showed that a proper balance between working and private life appeared to be a reason to work from home for some respondents, but a reason to not work from home for others. Also, WfH use may be related to long working hours and working in evenings and weekends, which, in turn, may have consequences for WHI and fatigue. Future (qualitative) research may be a fruitful way to gain more insight in underlying mechanisms related to the role of motives to (not) work from home as well as long working hours and working at unfavorable times in relation to wellbeing outcomes.

These findings fit the theoretical notions of individual differences in integration and segmentation preferences (16) and person-environment fit, which poses that individual wellbeing is optimal when characteristics of the context match those of the individual (17). Application of this theoretical notion to the context of WfH implies that a proper match between access to and need for WfH is important for employees to successfully and sustainably combine working life and private life. Our finding that an unfavorable WfH mismatch is associated with higher time-based WHI supports the time-regulation mechanism (13), meaning that an unfavorable WfH mismatch hampers employees to adapt working times to responsibilities in their private life. Besides, our findings that an unfavorable WfH mismatch is associated with strain-based WHI and fatigue are support for the recovery-regulation mechanism (13), meaning that WfH enables employees to adjust their working and resting times to their recovery needs.

### Methodological considerations

A strength of our study is that we used a large and heterogeneous sample of employees in The Netherlands. Nevertheless, two third of our population was male, which may be due to the fact that we excluded workers who had <24 contractual hours/week. The unequal male-female distribution may limit generalizability of the findings to some extent, but still our study sample included a group of 860 women. Also, post-hoc analyses showed no significant interactions between WfH mismatch and sex for all the outcomes, which indicates that findings of our study apply to men and women. Also, intermediate and high educational level were somewhat overrepresented. This may be a reflection of our choice to select participants who reported that their work lends itself to WfH, which is more common among people with higher compared to lower educational levels. Another strength is that that we used longitudinal data to replicate our cross-sectional findings, which allowed us to assess the temporal association between WfH mismatch and change in WHI and fatigue one year later. Nevertheless, the longitudinal analyses cannot provide clear evidence for causal links between WfH mismatch and WHI and fatigue. It is thus not clear whether a WfH mismatch causes WHI and fatigue or whether higher WHI and fatigue increase the need for WfH and as such a WfH mismatch. An analytical approach taking into account changes in WfH mismatch from baseline to follow-up would be better, but was not possible due to the high stability in WfH mismatch in our data, and, as a consequence, insufficient statistical power. Future research is recommended to gain more insight into causal links between WfH mismatch and wellbeing outcomes, including studies with a larger study sample with sufficient statistical power to study the effects of changes in WfH mismatch, as well as studies within settings in which changes in WfH mismatch can be expected, such as after changes in WfH policies or other reasons for intensification of WfH (eg, due to COVID-19). Another limitation of this study is that WfH need and WFH access have been measured with only one item each, and thus, WfH mismatch was based on two items. As WfH need and WfH access are relatively simple constructs, this might, however, not be problematic. In addition, WfH mismatch was treated as a dichotomous construct and we did standard linear regression analyses to gain insight in the association between WfH mismatch and WHI and fatigue. To explore the association in more detail, future research is recommended treating WfH mismatch as a continuous construct and to do more advanced statistical analyses, for example including polynomial regression analyses.

### Practical implications

Our results emphasize the need for tailored organizational WfH policies to reduce the risk of a person-environment misfit. Individual preferences with regard to WfH need to be taken seriously by organizations to prevent reduced employee wellbeing and protect sustainable employability. Prior to the COVID-19 pandemic, employers were sometimes reluctant to introduce a lenient WfH-policy, as they feared reduced work performance and suboptimal remote collaboration. However, these fears are found to be mostly unfounded in case WfH is supported by modern technology and clear performance targets (11). A tailored approach to WfH-policies implies some level of freedom in WfH; employees can evaluate whether WfH ≥1days a week fits their preferences and aligns with their personal situation (eg, a proper place to work at home) and work tasks at hand. The employer (ie, managers or supervisors) plays an essential role in reducing WfH mismatch and in securing tailored and thus favorable and sustainable WfH-policies.

### Working from home in the context of COVID-19

The COVID-19 pandemic catalyzed WfH, making this topic very relevant. However, WfH during our pre-COVID-19 data collection is different from WfH during the COVID-19 pandemic, which is very specific for several reasons (24). First, the COVID-19 outbreak made it impossible for employees and employers to anticipate large-scale WfH, and they had to abruptly move to fully working from home without relevant WfH-policies to manage this transition. Second, WfH became the (obligatory) default instead of the (self-chosen) exception and many employees had to work fulltime from home, instead of the one WfH-day a week that they may have had prior to the COVID-19 pandemic. At the same time, it may have increased the challenge to combine work and care responsibilities for many workers due to (temporarily) school closures. Although the results from the current study may not apply to WfH practices during the COVID-19 pandemic, the experiences from the pandemic may change how employers and employees perceive WfH. It is likely that WfH becomes an integral part of our future work society, maybe even in a more extensive form (ie, >1 day per week) than before the COVID-19 pandemic. Previously, employees may have had too little access to WfH in comparison to their need, whereas in future the opposite may be true with mandatory WfH even for those workers with a low need for WfH. Future research should address the effects of mandatory WfH on employees’ wellbeing.

### Concluding remarks

In conclusion, we found that an unfavorable mismatch between WfH access and need is associated with higher WHI and fatigue. A tailored WfH organizational policy, in which employees’ need for working from home is taken into account, may be a fruitful approach to utilize WfH as a way for employees to successfully and sustainably combine work and private life in its full potential.

### Funding

There was no funding source for this study.

### Conflict of interest

The authors declare no conflicts of interest.
